# Care transition from hospital to home: cancer patients’ perspective

**DOI:** 10.1186/s13104-020-05099-x

**Published:** 2020-06-01

**Authors:** Elisiane Lorenzini, Julia Estela Willrich Boell, Nelly D. Oelke, Caroline Donini Rodrigues, Letícia Flores Trindade, Vanessa Dalsasso Batista Winter, Michelle Mariah Malkiewiez, Gabriela Ceretta Flôres, Pâmella Pluta, Adriane Cristina Bernat Kolankiewicz

**Affiliations:** 1 Federal University of Santa Catarina, Florianópolis, SC Brazil; 2 University of British Columbia, Kelowna, BC Canada; 3 Regional University of the Northwest of the State of Rio Grande do Sul, Ijuí, RS Brazil

**Keywords:** Care transitions, Continuity of patient care, Measure, Neoplasms, Patient discharge, Patient readmission

## Abstract

**Objectives:**

The present database contains information on sociodemographic and clinical data as well as data from the Care Transition Measure (CTM 15-Brazil) of cancer patients undergoing clinical or surgical treatment. Data collection was carried out 7 to 30 days after patients’ hospital discharge from June to August 2019. Understanding these data can contribute to improving quality of care transitions and avoiding hospital readmissions.

**Data description:**

This data set encompasses 213 cancer patients characterized by the follow variables: gender, age range, place of residence, race, marital status, schooling, paid work activity, type of treatment, cancer staging, metastasis, comorbidities, main complaint, main complaint grouped as, continuing medication, diagnosis, diagnosis grouped as, cancer type, year of diagnosis, oncology treatment, first hospitalization, readmission in the last 30 days, number of hospitalizations in the last 30 days, readmission in the last 6 months, number of hospitalizations in the last 6 months, readmission in the last year, number of hospitalizations in the last year and the questions 1–15 from CTM 15-Brazil.

## Objective

Care transition (CT) is defined as a set of planned actions aimed at ensuring the coordination and continuity of care provided to patients, from admission to hospital discharge, and/or in the transfer of patients between units of the same location or different health services [1]. Further, it is understood as the time interval that begins with the preparation of the individual to be discharged from one service to another or being discharged home [2]. Continuity of care is one of the most significant challenges in health care services. Patients are attended by different professionals in various services of the health care network, requiring integration and connectivity of care over time [3]. In view of this scenario, cancer patients are part of the group of patients affected by chronic diseases, who need continuous care throughout the network care. Cancer patient’s hospital discharge requires commitment from the team, patient and family. CT is being considered an important strategy to contribute to continuity of care, reducing the number of hospital admissions, as well as impacting the decrease in readmissions caused by complications [4]. Furthermore, this strategy may reduce the cost of health services, contribute to more effective treatment of patients, the longevity of people with comorbidities and improve the quality of life for patients and their families [5–8]. The present database contains relevant information about cancer patients’ discharge and CTs. Understanding the variables of the CTM and its relation with cancer patients’ characteristics may contribute to developing effective strategies for the continuity of care.

## Data description

These data were collected for a cross-sectional study, developed with cancer patients undergoing clinical or surgical treatment, after hospital discharge during the period of 7 to 30 days in a philanthropic Hospital in the Northwest of the State of Rio Grande do Sul, Brazil. All cancer patients aged 18 years or older who were hospitalized for at least 24 h were included. Patients unable to answer the questionnaire and who were not accompanied by caregivers or family members were excluded (8 patients). The patient was invited to participate in the study during hospitalization; those who agreed to participate signed the consent form as approved by the hospital’s Ethics and Human Research Committee. After hospital discharge, the patient was contacted by telephone to answer the questionnaires. When collecting data from the CTM-15, by telephone, those who did not answer the calls after the third attempt were considered missing, within seven to 30 days after hospital discharge (10 losses). Data collection was carried out between the months of June and August 2019, using the sociodemographic and clinical questionnaires and the Care Transitions Measure instrument (CTM 15-Brazil) [9]. The CTM 15-Brasil was adapted and validated in 2016, being the first Brazilian instrument used to analyze the quality of CTs for inpatients discharged home. This survey measures and evaluates, from the patient’s perspective, the quality of the transition from hospital discharge to home or between different health services. It consists of 15 items, organized into four factors: health management preparation; medication understanding; important preferences, and care plan. It is usually applied by telephone and allows the caregiver/familiar to answer as a proxy informant if the participant has a very compromised health condition [9]. This data set encompasses 213 patients characterized by the following variables: gender, age range, place of residence, race, marital status, schooling, paid work activity, type of treatment, cancer staging, metastasis, comorbidities, main complaint, main complaint grouped as, continuing medication, diagnosis, diagnosis grouped as, cancer type, year of diagnosis, oncology treatment, first hospitalization, readmission in the last 30 days, number of hospitalizations in the last 30 days, readmission in the last 6 months, number of hospitalizations in the last 6 months, readmission in the last year, number of hospitalizations in the last year and the questions 1–15 from CTM 15-Brazil. The analysis of this data can be performed by using descriptive statistics and measures of central tendency (mean and median) and variability (standard deviation and interquartile range), as well as absolute and relative distributions (n−%). The symmetry of continuous distribution can be assessed by the Kolmogorov–Smirnov test. The comparison of continuous variables between two or more independent groups can be performed using the Variance Analysis technique—ANOVA (One Way)—Post Hoc Tukey (independent groups of similar sizes) or Scheffé (independent groups of very different sizes and/or heterogeneity variances), for example. Also, multivariate logistic regression analysis, odds ratio and relative risk analyses can be performed (Table 1).

**Table 1:** Overview of data file

Label	Name of data file/data set	File types (file extension)	Data repository and identifier (DOI or accession number)
Data file 1	Cancer patient’s care transition database	Cancer patient’s care transitions dataset.xlsx.	10.6084/m9.figshare.11831343.v3 [10]

## Limitations

The limitation of this data set is its restriction to a local single hospital. However, it remains relevant as this field is still unexplored in Brazil. This data is the first of its kind from Brazil to be available online. There is no national policy on CTs in Brazil. Researchers who are interested in CTs of cancer patients can extensively explore the variables described here.

## Data Availability

Lorenzini, Elisiane; Boell, Julia Estela Willrich; Oelke, Nelly D.; Rodrigues, Caroline Donini; Trindade, Letícia Flores; Winter, Vanessa Dalsasso Batista; et al. (2020): Cancer patient´s care transition database.xlsx. figshare. Dataset [10]. 10.6084/m9.figshare.11831343.v3.

## Abbreviations

CT: Care transition
CTM: Care Transitions Measure

## References

[1] Coleman EA, Boult C (2003). Improving the quality of transitional care for persons with complex care needs. J Am Geriatr Soc.

[2] Acosta AA, Lima MADS, Pinto IC, Weber LAF (2020). Care transition of patients with chronic diseases from the discharge of the emergency service to their homes. Rev Gaúcha Enferm..

[3] Oikonomou E, Chatburn E, Higham H (2019). Developing a measure to assess the quality of care transitions for older people. BMC Health Serv Res.

[4] Lima MADS, Magalhães AMM, Oelke ND, Marques GQ, Lorenzini E, Weber LAF (2018). Care transition strategies in Latin American countries: an integrative review. Rev Gaúcha Enferm..

[5] Flemming MO, Haney TT (2013). Improving patient outcomes with better care transitions: the role for home health. Cleve Clin J Med..

[6] Flink M (2018). Measuring care transitions in Sweden: validation of the care transitions measure. Int J Qual Health Care.

[7] Wakiuchi J, Marcon SS, Oliveira DC, Sales CA (2019). Chemotherapy under the perspective of the person with cancer: a structural analysis. Texto Contexto Enferm..

[8] Rohsig V, Silva P, Teixeira R, Lorenzini E, Maestri R, Saraiva T (2019). Nurse navigation program: outcomes from a breast cancer center in Brazil. Clin J Oncol Nurs..

[9] Acosta AM, Lima MADS, Marques GQ, Levandovski PF, Weber LAF (2017). Brazilian version of the Care Transitions Measure: translation and validation. Int Nurs Rev.

[10] Lorenzini E, Boell JEW, Oelke ND, Rodrigues CD, Trindade LF, Winter VDB, et al. Cancer patient’s care transition database.xlsx. figshare. Dataset. 2020. 10.6084/m9.figshare.11831343.v3.

